# Interpretation of ambiguous trials along with reasoning strategy is related to causal judgements in zero-contingency learning

**DOI:** 10.1177/17470218231155897

**Published:** 2023-02-24

**Authors:** Gaëtan Béghin, Henry Markovits

**Affiliations:** Université du Québec à Montréal, Montreal, QC, Canada

**Keywords:** Contingency learning, individual differences

## Abstract

The dual strategy model suggests that people can use either a Statistical or a Counterexample reasoning strategy, which reflects two qualitatively different ways of processing information. This model has been shown to capture individual differences in a wide array of tasks, such as contingency learning. Here, we examined whether this extends to individual differences in the interpretation of contingency information where effects are ambiguous. Previous studies, using perceptually complex stimuli, have shown that the way in which participants interpret ambiguous effects predicts causal judgements. In two studies, we attempted to replicate this effect using a small number of clearly identifiable cues. Results show that the interpretation of ambiguous effects as effect present is related to final contingency judgements. In addition, results showed that Statistical reasoners had a stronger tendency to interpret ambiguous effects as effect present than Counterexample reasoners, which mediates the difference in contingency judgements.

Human reasoning is characterised by variability. There is now considerable evidence in a variety of reasoning and judgement tasks, that for normatively equivalent problems, people will not produce equivalent answers (e.g., Evans et al., 1983; Kahneman & Tversky, 1973; Newstead et al., 1992; Tversky & Kahneman, 1974). For example, in the context of deductive reasoning, belief bias effect shows that for deductive inferences where the logical validity of a putative conclusion conflicts with its empirical truth, people will not systematically choose the valid response (Evans et al., 1983). Thus, to better understand human reasoning, it is important to identify the nature of individual differences that underlie such variability. In the following, we examine a recent model of individual differences, the dual strategy model of reasoning, and how this applies to understanding differences in the context of contingency learning with ambiguous effects.

The dual strategy model of reasoning was based on an initial proposal by Verschueren et al. (2005) and subsequently modified in a series of studies (e.g., Markovits et al., 2013, 2015; Thompson & Markovits, 2021). This model suggests the existence of two different reasoning strategies: a *Counterexample* strategy, based on mental model theories (Johnson-Laird, 2001; Johnson-Laird & Byrne, 2002), and a *Statistical* strategy, based on probabilistic theories (Oaksford & Chater, 2009). People using a Statistical strategy tend to process information regarding a conclusion or a judgement in a broad and intuitive way to generate a rapid likelihood estimation of a potential conclusion being true. People using a Counterexample strategy tend to focus on a smaller range of important information to construct a mental representation of key problem parameters and are particularly sensitive to potential counterexamples. One clear example of such a difference is illustrated in results obtained by Markovits et al. (2018). In this, people were presented with simple inferences of the form “If P then Q, P is true. Is Q true?” They were also presented with observational data indicating the relative ratio of cases of P and Q and of P and not-Q. This went from 100:0 to 99:1 to 75:25. This ratio determines two parameters. On the one hand, a higher ratio corresponds to a greater probability of the putative conclusion (i.e., that Q is true if P is true). On the other hand, any ratio less than 1 indicates the existence of potential counterexamples to this same conclusion. Statistical reasoners conclude that “Q is true” at a rate that closely maps the probability that the conclusion is true. Counterexample reasoners accept the 100:0 conclusion at the same rate as Statistical reasoners, but uniformly reject the conclusion if there are any potential counterexamples, that is, for both the 99:1 and 75:25 conditions. In other words, these strategies correspond to qualitatively different ways of processing the same information.

Initial studies resulted in the creation of a Strategy Assessment Task (Markovits et al., 2012, 2013), which allows categorising participants according to their preferred strategy. The key items consist of 10 Affirmation of the Consequent (AC) conditional inferences (i.e., If P then Q, Q is true. Is P true?) constructed with abstract content, situated on an imaginary planet. Participants are asked to evaluate the validity of the conclusion that “P is true.” Each inference is associated with explicit observational information regarding the relative occurrence of cases in which both P and Q occur together, and cases in which not-P and Q occur together. The ratio of P&Q cases to not-P&Q cases determines the empirical probability of the conclusion being true. Of the 10 AC inferences, 5 were chosen so that this probability was very high, but less than certain. In these (90%) cases, people were told that the ratio was close to 90:10 (of 1,000 observations, 900 corresponded to P&Q, while 100 corresponded to not-P&Q). For the other 5 (50%) inferences, the ratio was 50:50. Critically, both sets of inferences present cases of not-P&Q, that is, they all indicate the presence of potential counterexamples to the conclusion that “P is true.”

This allows clearly distinguishing two ways of processing the information included with these inferences. People who compute the relative probability of the conclusion based on the given information (*Statistical* reasoners) should accept the conclusion that “P is true” more often with 90% inferences than with 50% inferences, in a way that reflects the difference in relative probabilities. People who concentrate on potential counterexamples (*Counterexample* reasoners) should reject this conclusion for all the inferences. Previous studies examining the dual strategy model have focused on the difference between Statistical and Counterexample reasoners, which typically categorise between 65% and 75% of participants. However, it should be noted that a recent large-scale study (Thompson & Markovits, 2021) reported that participants not categorised as either Statistical or Counterexample could be categorised into two further classes: Intermediate participants who switched between the Counterexample and Statistical strategies, and those who did not understand the nature of the task. More specifically, Intermediate reasoners appear to generally use a Counterexample strategy with occasional lapses into a more statistical approach (Thompson & Markovits, 2021). Previous results suggest that these reasoners produce performance levels that are somewhat lower than Counterexample reasoners but higher than Statistical reasoners. The Other category designates participants with lower cognitive abilities (i.e., lower IQ), which is associated with more difficulties in understanding the task and producing consistent responses to problems. This latter category produces results that are lower or equal to those of Statistical reasoners. Now, the clearest distinction that is made by the dual strategy model is that between Counterexample and Statistical reasoners, one which is supported by a variety of studies. In the present studies, we concentrate on this distinction.^1^

Several studies have shown that the distinction between Statistical and Counterexample strategies is a robust predictor of individual differences in a variety of reasoning and judgement tasks (Markovits et al., 2012, 2013, 2015; Thompson & Markovits, 2021). Furthermore, other studies have shown that strategy use captures individual differences in a much larger array of contexts, such as emotion processing (Markovits et al., 2018), mental rotation (Markovits, 2019), social biases (Gagnon-St-Pierre et al., 2021), and acceptance of Fake news (Gratton & Markovits, 2021). These studies suggest that the dual strategy model not only predicts deductive reasoning performance but also captures some key differences in the way that information is processed.

One such context is that of the use of contingency information in causal learning. There is a great deal of evidence that shows that people do use contingency information to generate judgements of causality (Hattori & Oaksford, 2007; Perales & Shanks, 2007, for overviews). Such judgements occur in many different practical contexts, such as medical diagnoses, and it is both theoretically and practically important to understand the kinds of individual difference factors that could affect the way that people use contingency information. Although many studies have suggested the existence of individual differences, they have not provided any way of distinguishing these differences. However, the dual strategy model has recently been shown to be a useful way of predicting individual differences in classical contingency learning paradigms. An initial study showed that Statistical reasoners, who use a more intuitive mode of processing, tend to focus more strongly on sufficiency information, that is, the ratio of positive outcomes when a putative cause is present, while Counterexample reasoners put more weight on necessity information, that is, the ratio of positive outcomes in the absence of a putative cause (Béghin et al., 2021). This difference leads to the general prediction that, all other things being equal, contingency information will produce higher ratings of efficacy among Statistical than among Counterexample reasoners. This latter hypothesis was indeed confirmed in a recent study Béghin and Markovits (2022) in which the effect of strategy use was examined in the context of manipulation of the plausibility of the putative cause.

In the following studies, we wished to extend the analysis of individual differences in contingency learning to a relatively new paradigm. Most studies of contingency learning have used binary variables to represent the presence/absence of a putative cause and the presence/absence of an effect. However, recently, some researchers have called for more ecological experiments, arguing that binary variables do not truly reflect real-world situations. Although this applies to both causes and effects, these studies have concentrated on the way that effects are represented (Chow et al., 2019; see also Soo & Rottman, 2018, for the use of continuous variables to study causal induction). They argued that in many real-world situations, outcomes are variable and may be ambiguous, which is not reflected with binary outcomes (outcome present/absent). Chow and colleagues (2019) studied outcome density bias using a continuous rating of the outcome. They asked participants to evaluate the efficacy of a drug to cure a disease, but instead of presenting binary outcomes (e.g., whether the patient is cured or not), they used a continuous outcome (e.g., improvement of a patient from taking a medicine on a scale from 0 to 100). In two studies using continuous outcomes, they reported a systematic density bias, comparable to that found with binary outcomes.

Blanco et al. (2020) extended this work using visually complex representations. They presented patients’ states using images representing human tissues with 2,500 dark and light cells. The ratio of dark to light cells determined the extent to which a patient was ill or not. When the number of light cells was superior to the number of dark cells (e.g., 90/10, 80/20, 60/40), the patient was relatively well. When the ratio was inverted, the patient was relatively ill. Participants were given explicit instructions about the way to interpret these cases and during the learning procedure were asked to indicate for each patient whether the cue showed that the patient was well or not. However, their main focus was on ambiguous trials, that is, trials where there were an equal number of light and dark cells. The key study was one in which the normative contingency, computed over non-ambiguous trials, was null ( $\Delta_{p}=0$ ).

Their results showed that when contingency was null, participants’ interpretation of the ambiguous trials uniquely predicted their final causal judgements. Specifically, there was a positive relation between the extent to which ambiguous cues were interpreted as indicating that patients were well and higher judgements of the efficacy of the treatment. These results suggest that an individual’s tendency to interpret ambiguous outcomes as indicating a state of wellness is associated with a higher estimate of the efficacy of an objectively ineffective treatment.

As Blanco et al. (2020) remarked, there are clear individual differences in the extent to which people generate such interpretations. Thus, to better understand how humans process contingency information, it seems important to understand what factors characterise this variability. One of the aims of this study was to determine whether the dual strategy model provides a framework to analyse individual differences in this context. The clearest prediction relies on previous results that show that when given the same contingency information (i.e., equal number of trials supporting the presence and the absence of a causal relationship), Statistical reasoners tend to put more weight on positive compared with negative information in contingency judgements (Béghin & Markovits, 2022). Also, studies in the context of deductive reasoning showed a similar pattern of results, that is, when given with both positive and negative evidence supporting a conclusion, Statistical reasoners tend to accept the conclusions more frequently than Counterexample reasoners (Brisson & Markovits, 2020; Markovits et al., 2018; see also Gratton & Markovits, 2021, for a similar pattern of results in the context of repetition and disconfirmation on the believability of Fake news). Given the fact that this pattern of responses is generalised across tasks, and more fundamentally reflects a difference in processing between Strategies, we expected to find difference between Strategies in the categorisation of ambiguous trials. In fact, cues of the kind examined by Blanco et al. (2020) are composed of both negative and positive information about the presence of the effect. Thus, the most straightforward prediction would expect that Statistical reasoners would show a greater tendency to interpret ambiguous stimuli as indicating a state of wellness, by weighting the positive information more strongly than the negative information.

The second aim of these studies was to generalise the results of the Blanco et al. (2020) study to a simpler set of cues. In this study, cues to patient’s states were perceptually complex: For each image, there were 2,500 cells, each of which provided a colour-coded indicator of patient well-being. Participants had to rely on their perceptual impressions to interpret the image because it was impossible to calculate the real numbers of positive and negative cues. This was done to simulate a situation in which clinicians might be faced with a very large quantity of data, with the explicit goal of seeing whether there was a tendency to perceive patterns in complex data when there were none. In fact, Blanco et al. (2020) interpreted their results as explicable by the operation of pattern recognition processes. However, in many cases there are more limited sets of clinical data, and explanations in terms of pattern recognition processes are not required (i.e., presence of specific and limited sets of physical or mental symptoms). One less constrained interpretation of Blanco et al.’s (2020) results would hypothesise that individual differences in the evaluation of ambiguous cues (and their effect on causal judgements) would extend to cues even with limited quantities of information, which would not require any form of pattern recognition.

## Study 1

To more specifically examine this hypothesis, we decided to use a very small number of cues that were easy to decode. We also introduced one other variation in the method used by Blanco et al. (2020). To limit effects of cue ambiguity to the single case where there were identical numbers of positive and negative cues, participants were given explicit instructions about how to interpret outcomes, which stated that the presence of a preponderance of negative or positive cues indicated that the patient was ill or well, respectively. However, in real-life situations, ambiguity is not only restricted to cases with an equal number of positive or negative cues, and any tendency to give more weight to positive information might apply equally well to cues with partial levels of ambiguity. To examine this possibility, we gave no specific instructions about how to interpret cues with a mix of positive and negative information.

Thus, in the following study, we gave participants a null Contingency Learning Task where patient state was represented by a discrete set of cues, each of which presented the state of four different clinical categories. Individual cues were either good (smiley green face) or bad (frowning red face) images. For each trial, the state of the patient was represented by different combinations of cues, going from all of one kind to completely ambiguous (equal number of good and bad images). As in Blanco et al. (2020), participants were asked to categorise each set of cues as indicating that the patient was well or not, but were given no instructions about how to interpret patient states. Participants were also given the Strategy Assessment Task used to determine reasoning strategy. We specifically hypothesised that Statistical reasoners would show a greater tendency to interpret ambiguous cues as indicating a state of wellness than Counterexample reasoners. We also expected to replicate the previous results of Blanco et al. (2020), that is, that there would be a positive relation between the tendency to interpret ambiguous cues as indicating a state of wellness and final causal attributions.

### Data availability

Data for these studies are available at https://osf.io/cw2a8/?view_only=4baf1f3a486b43758a39bc36da064c50.

### Method

These projects were approved by the Institutional Review Board (IRB) of the Université du Québec à Montréal (approval number: 1068-2020-1681). All participants provided informed consent.

#### Participants

A total of 251 participants (163 females, 88 males, mean age = 30.4 years) were recruited from the online participant pool Prolific. Prolific is a recruitment site for on-line studies based in the United Kingdom which allows linking a study to participants from more than 30 different countries. The strategy diagnostic task usually categorises between 65% and 75% of participants in either the Counterexample or Statistical strategy. The stopping rule was designed to leave around 150 participants who were classed as Statistical or Counterexample reasoners, which is sufficient to detect a medium effect ( $\eta_{p}^{2}=.05$ ) with a probability of .8. Participants each received £1.5.

### Material

#### Strategy Assessment Task

The Strategy Assessment Task consisted of a set of 13 inferences identical to those used in previous studies (Markovits et al., 2012). Participants were told that they were to be given information about a newly discovered planet called “Kronus.” They were asked to evaluate the logical validity of a sequence of premises followed by a conclusion based on previous information. The set of 13 items described conditional statement including explicit frequency information about the relative occurrence of the antecedent and the consequent. Among the 13 inferences, 3 were included as fillers and were modus ponens of the form P implies Q, P is true. The 10 others were *AC* of the form P implies Q, Q is true. Among the 10 inferences, 5 were associated with statistical information indicating a high probability of P being true, close to 90%. The others were associated with information indicating a 50% probability that P was true. All the items of the task along with the instructions to compute the reasoning Strategy are available in Supplementary Material 1.

#### Contingency Learning Task

In this task, participants were told to imagine that they were a psychologist specialised in a rare mental disorder called “Hylophobia” and that they wanted to test for the efficacy of a 12-week protocol that they developed to cure this disorder. Participants were informed that they were going to see the results of their experiment in which the protocol was tested on 52 patients who were suffering from this disorder and were asked to evaluate the efficacy of the protocol to cure “Hylophobia.” Specific instructions are available in Supplementary Material 1.

The learning task consisted of 52 trials, each of which presented the final state of a single patient, some of which received the protocol and others did not. This was measured by four indicators of different classes of symptom, named, respectively, A, B, C, and D. For each of these four classes, participants were given one of two kinds of cues. A green smiling face indicated that this set of symptoms was resolved, while a red sad face indicated that the symptoms were not resolved. Thus, for each trial participants were informed whether the patient had received the treatment or not and were presented with the four indicators of the patient’s current state. Based on that information, participants were asked to evaluate whether the participant was cured or not using one of two buttons (“The patient is cured,” “The patient is not cured”).

**Figure 1.**
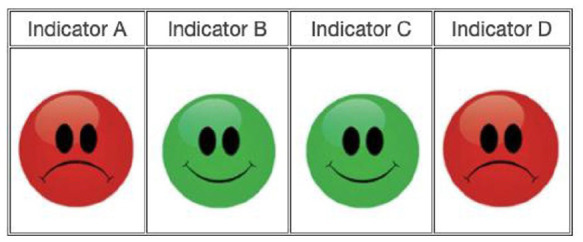
Example of an ambiguous effect in the Contingency Learning Task in Study 1.

Presented cues used one of five different combinations of the indicators. Among them, two showed more positive than negative cues (i.e., positive ratio of evidence): four green smiling faces (4:0), three green smiling faces, and one red sad face (3:1); two showed more negative than positive cues (i.e., negative ratio of evidence): four red sad faces (0:4), three red sad faces, and one green smiling face (1:3). The final combination which presented two green smiling faces and two red sad faces was completely ambiguous because it contained equal number of positive and negative information (2:2) (Figure 1). For combinations having both kinds of faces, the positions of the faces were counterbalanced, that is, for each specific combination of red and green faces, another was constructed where the positions of the red and green faces were inverted.

Before the learning trials, participants were presented with two examples to better understand the meaning of each of the cues. One presented a trial with all indicators exhibiting a green smiling face, and the other example exhibited four sad faces. It should be noted that participants were not instructed about the effects of combinations of cues. Examples are available in Supplementary Material 1.

The 52 cues presented on the 52 trials consisted of 20 completely ambiguous trials (2:2). The remaining trials consisted of 16 trials with more positive than negative evidence (4:0; 3:1) and 16 trials with more negative than positive evidence (1:3, 0:4); each combination was present at the same rate (i.e., eight times). In addition, the rate at which the treatment was given or not was the same among all trials. Thus, if trials with a positive or negative ratio are categorised consistently with that information, the contingency computed based on those trials was null ( $\Delta_{p}=0)$ . Combinations’ parameters are synthesised in Table 1.

**Table 1.** Combination parameters in Study 1.

	Negative ratio		Equal ratio	Positive ratio	
Ratio (smiling faces:sad faces)	0:4	1:3	2:2	3:1	4:0
Cause present	4	4	10	4	4
Cause absent	4	4	10	4	4

At the end of the learning phase, participants were asked to judge the efficacy of the treatment using a scale from 0 (“Definitely No”) to 100 (“Definitely Yes”). The question asked was as follows: “To what extent do you think that the protocol reduced participants’ symptoms of Hylophobia.”

#### Procedure

The order of the two tasks was counterbalanced (Contingency Learning Task, Strategy Assessment Task). The order of the trials in the Contingency Learning Task was randomised.

### Results

#### Categorisation of trials

First, we examined participants’ categorisation of patient state on each trial as indicating that the patient was cured or not. We computed a Generalised Mixed Model (using lme4 package for R [Bates et al., 2015; R Core Team, 2021], with emmeans [Lenth et al., 2022] for contrast analysis with Holm’s correction) using a binary logistic regression with the categorisation of each trial (0 = patient not cured, 1 = patient cured) as the dependent variable and number of green Faces (as an ordinal variable) and Cause (Absent, Present) along the interaction between these variables as independent variables. We used Participant and Items as random effects to account for the repeated measures (the model was Categorisation ~ Face × Cause + (1|Id) + (1|Item)). This gave a significant main effect for Faces, $\chi^{2}$ (1, *N* = 251) = 462.96, *p* < .001, and Cause, $\chi^{2}$ (1, *N* = 251) = 5.89, *p* = .015. Also, the Cause × Faces interaction was significant, $\chi^{2}$ (1, *N* = 251) = 4.37, *p* = .037.

The number of green faces positively predicted the level of categorisation of trials as patient cured, that is, as the number of green faces increased, categorisation of trials as patient cured also increased. Participants significantly categorised more trials as patient cured when the putative cause was present (*EMM* = 0.15, *SE* = 0.03) than when it was absent (*EMM* = 0.08, *SE* = 0.02).

To examine the interaction effects, we conducted contrast analyses (*z*-test) using emmeans for R (Holm’s correction, *p* = .05). To analyse the Cause × Faces interaction, we conducted a simple slope test (using interactions package for R (Long, 2021) and then used a contrast analysis (emmeans) to compare these slopes. Results showed that both when the putative cause was absent and present, the number of green faces positively predicted the level of categorisation (Cause present: *b* = 3.00, *z* = 16.65, *p* < .001, Cause absent: *b* = 2.49, *z* = 14.49, *p* < .001). Contrasts analysis showed that the number of green faces was significantly more correlated to the level of categorisation when the cause was present than when it was absent.

We then examined whether reasoning strategy predicted individual differences in the categorisation of trials. We first analysed results from the Strategy Assessment Task. Previous studies have concentrated on the distinction between Statistical and Counterexample reasoners (Markovits et al., 2012, 2013). However, Thompson and Markovits (2021) provided evidence for the existence of two other categories: an Intermediate category composed of people who are mostly using a Counterexample strategy, but are changing between a Statistical strategy, and an Other category, composed of people who have difficulty in understanding the basic structure of the tasks. Although we still concentrated on the Counterexample/Statistical distinction, we analysed results using the extended categories, using the following criteria. Note that the Strategy assessment instrument is composed of five inferences for which there was a high probability of the putative conclusion being true (90% items) and five inferences for which this probability was relatively low (50% items). Participants who rejected all of the high (90%) and low (50%) probability inferences were classified as Counterexample reasoners. Participants who rejected the 50% inferences at least two times more than the 90% inferences were classified as Statistical reasoners. Participants who rejected four of the five 90% inferences were classified as Intermediate reasoners. All others were classified as Other. This gave 104 statistical reasoners, 53 counterexample reasoners, 34 Intermediate reasoners, and 60 Other reasoners. In the following, we report the full analyses, but post hoc comparisons focus only on the Counterexample/Statistical distinction. Intermediate reasoners are assumed to change strategies, and thus their results should fall between those of Counterexample and Statistical reasoners. Other participants do not understand the basic tenet of tasks. These participants are associated with lower cognitive abilities and are less consistent in their responses, and thus should be more prone to biases. Specific comparisons including the Intermediate and Other categories are reported in Supplementary Material 2.

We then computed a Generalised Mixed Model (using lme4 package for R, emmeans for contrast analysis with Holm’s correction) using a binary logistic regression with the Categorisation of each trial (0 = patient not cured, 1 = patient cured) as the dependent variable and Strategy (Counterexample, Statistical, Intermediate, Other), number of green Faces, and Cause along with their interaction as independent variables. We included Participant and Item as random effects to account for the repeated measures (the model was Categorisation ~ Strategy × Faces × Cause + (1|Id) + (1|Item)).

This gave significant main effects of Faces, $\chi^{2}$ (1, *N* = 251) = 497.19, *p* < .001, Strategy, $\chi^{2}$ (3, *N* = 251) = 20.98, *p* < .001, and Cause, $\chi^{2}$ (1, *N* = 251) = 7.07, *p* = .009. The Strategy × Faces, $\chi^{2}$ (3, *N* = 251) = 80.53, *p* < .001, Strategy × Cause, $\chi^{2}$ (3, *N* = 251) = 24.96, *p* < .001, and Strategy × Ratio, $\chi^{2}$ (3, *N* = 251) = 24.37, *p* < .001, interactions were significant. The three-way Cause × Strategy × Faces interaction was also significant, $\chi^{2}$ (3, *N* = 251) = 20.98, *p* < .001.

As in the first analysis, the number of green Faces positively predicted categorisation of trials as patient cured. The main effect of Cause showed that the estimated marginal mean of categorisation of trials as patient cured was significantly higher when the cause was present (*EMM* = 0.12, *SE* = 0.02) than when it was absent (*EMM* = 0.08, *SE* = 0.2). The main effect of Strategy showed that the estimated marginal mean of categorisation of trials as patient cured was significantly higher for Statistical reasoners (*EMM* = 0.14, *SE* = 0.03) than for Counterexample reasoners (*EMM* = 0.02, *SE* = 0.006).

Analysis of the Cause × Strategy interaction showed that when the cause was both present and absent, Statistical reasoners categorised significantly more often patient as cured than Counterexample reasoners (Counterexample, Cause absent: *EMM* = 0.01, *SE* = 0.004, Cause present: *EMM* = 0.03, *SE* *=* 0.01; Statistical, Cause absent: *EMM* = 0.12, *SE* *=* 0.03, Cause present: *EMM* = 0.17, *SE* = 0.04). For both strategies, this type of categorisation was not significantly different between cause present and absent, although this difference was greater among Statistical reasoners.

**Figure 2.**
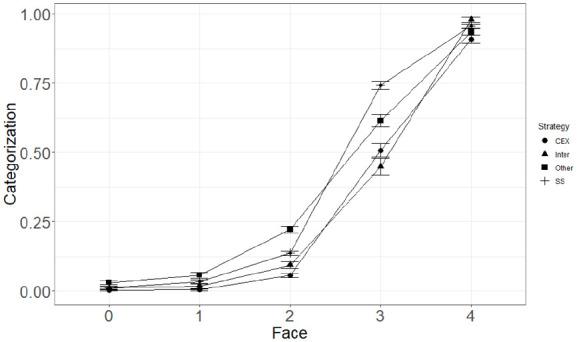
Mean levels of Categorisation as a function of the number of green Faces and the Reasoning Strategy (CEX, Inter, Other, SS).

Analysis of the Strategy × Faces interaction showed that the number of green faces significantly predicted level of categorisation of trials as patient cured for both Counterexample and Statistical reasoners. However, the number of green faces was more strongly correlated with the level of categorisation for Counterexample (*b* = 4.11, *SE* = 0.25) reasoners than for Statistical reasoners (*b* = 2.97, *SE* = 0.14). Results from the three-way interaction did not show any significant divergence from the two-way interactions reported previously. However, this overall difference cannot be clearly interpreted without examining the specific differences for each level of positive and negative cues. Thus, we conducted pairwise comparisons of categorisation between Strategies for each level of Faces and Cause (Wilcoxon test, with Holm’s *p* value adjustment—see Figure 2). These showed that when all faces were red and when there was one green face, levels of categorisation were similar between Counterexample and Statistical reasoners, both when the putative cause was present and absent. However, when there were two green faces (i.e., truly ambiguous trials), Counterexample reasoners categorised significantly less often patient as cured than Statistical reasoners; this was true both when the putative cause was present and absent. For trials with three green faces, Statistical reasoners had higher levels of categorisation of trials as patient cured than Counterexample reasoners when the putative cause was present. However, no difference was observed when the putative cause was absent. Finally, for trials with all faces being green, categorisations of trials were similar between Counterexample and Statistical reasoners when the cause was both absent and present.

#### Effect of categorisation on judgements

We then examined the influence of the way that patient state was categorised on final judgements of efficacy. We first computed the proportion of all trials that participants categorised as patient cured (referred to as subjective P(O) following Blanco et al., 2020). We then calculated the actual contingency. As participants varied in the way that they categorised patient state, they necessarily varied in the specific contingency information they received. Thus, following Blanco et al. (2020), we categorised each trial into one of four types of events. A trial was Type a when the treatment was received and the patient was judged as cured; Type b when the treatment was received and the patient was judged as not cured; Type c when the treatment was not received and the patient was judged as cured; and Type d when the treatment was not received and the patient was judged as not cured. Based on these values, we computed the subjective contingency for each participant using the standard formula for $\Delta_{p}.$ .

**Figure 3.**
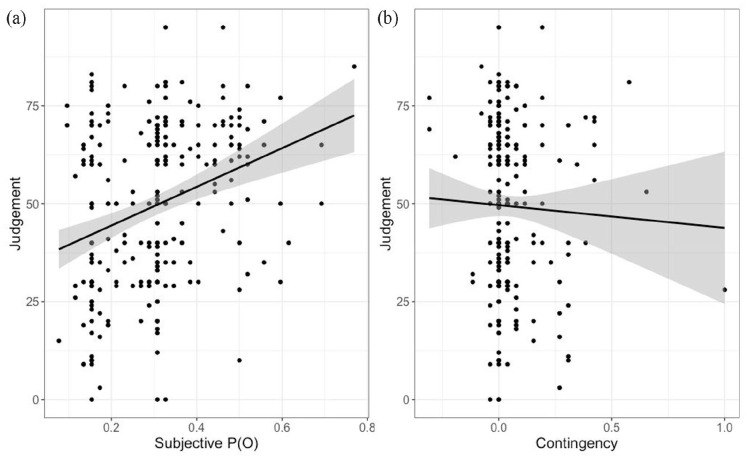
Scatterplot of judgements as a function of subjective P(O) (a) and contingency (b) in Study 1.

We then computed a linear regression with Judgement as the dependent variable and Contingency and subjective P(O) as independent variables. This gave a significant effect for subjective P(O), *b* = 49.82, *t*(248) = 5.1, *p* < .001. Results for contingency were not significant, *b* = −8.51, *t*(*48*) = −0.87, *p* *=* .387. This shows that Judgements of efficacy were strongly correlated with subjective P(O), and not at all related to contingency, consistent with the results of Blanco et al. (2020; see Figure 3).

We then examined whether the Counterexample/Statistical difference in strategy use affected the relation between categorisation of trials and final judgements of efficacy. We first conducted a linear regression with judgement of efficacy as the dependent variable and Strategy and Contingency as independent variables. This gave a significant effect of the contrast between Statistical and Counterexample reasoners, *b* = 9.54, *t*(246) = 2.7, *p* = .007. Results for Contingency were not significant, *b* = −13.74, *t*(247) = −0.772, *p* = .44. We examined this effect by conducting post hoc analysis (Tukey’s correction, *p* = .05). Judgements of efficacy were higher among Statistical (*M* = 50.8, *SD* = 20.5) than among Counterexample reasoners (*M* = 41.2, *SD* = 21.5).

**Figure 4.**
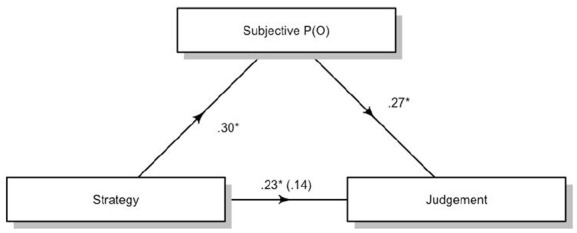
Standardised regression coefficients for the relationship between Strategy (Counterexample and Statistical reasoners only) and Judgement as mediated by Subjective P(O) in Study 1. The coefficient between Strategy and Judgement while controlling for Subjective P(O) is in parentheses. *p < .05.

We then conducted a mediation analysis using judgement of efficacy as the dependent variable, Strategy as the independent variable, and subjective P(O) as the mediator. We used the JAMOVI (The jamovi project, 2021) package for mediation analysis, which is an adaptation of the R Lavaan package (Gallucci, 2020; Rosseel, 2019). We used bootstrapping with 5,000 samples to test for the indirect effects. Results showed that the indirect effect of subjective P(O) was significant, suggesting that subjective P(O) mediates the difference in judgement between these strategies, *b* = 3.49, 95% confidence interval (CI) = [1.27, 5.68]. The residual direct effects were still significant, *b* = 9.64, 95% CI = [2.80, 16.47]. Figure 4 synthesises the mediation model for the effects of the contrast for counterexamples and statistical reasoners.

### Discussion

In this study, participants were given a set of four cues to patient state, which were positive or negative, with no explicit instructions how to interpret states with both kinds of cue. The results of this study replicate the key finding of Blanco et al. (2020). We found the same strong relation between the tendency to interpret ambiguous trials as indicating that a patient was cured and final judgements of efficacy. In our study, ambiguity was not only restricted to trials with an equal number of positive and negative information, but its effect was broadened to any cues with some combination of negative and positive information. However, unlike Blanco et al. (2020), we found that the presence or absence of the putative treatment did influence the way that cues were categorised. Participants tended to categorise trials more often as patient cured when the putative treatment was present than when it was absent. In addition, the relation between the relative quantity of positive information and the tendency to categorise cues as patient cured was stronger when the putative treatment was present than when it was absent.

Finally, these results show that Statistical reasoners showed a stronger tendency to categorise trials as patient cured than Counterexample reasoners. This difference in categorisation of ambiguous and positive trials mediated the Counterexample/Statistical difference in judgement of efficacy.

## Study 2

The results of Study 1 replicate the key result of Blanco et al. (2020) showing that participants who tend to interpret ambiguous cues as indicating that a patient is cured tend to overestimate the efficacy of an objectively ineffective treatment. They also show that strategy use is a good predictor of the extent to which people interpret ambiguous cues positively. However, in contrast with the results of Blanco et al. (2020), they also show that such positive interpretations are more frequent when the putative treatment is present. They also suggest that the increased rate of positive interpretations found with Statistical reasoners is relatively stronger when the treatment is present compared with when it is absent. In this study, we aimed to both generalise the results of Blanco et al. (2020) and examine the source of the discrepancy between their results and those of Study 1. Specifically, it is possible that the discrepancy between Study 1 and the results of Blanco et al. (2020), which is shown in the effect of presence and absence of the putative cause, might be due to the use of very simple cues used to represent the effect. This suggests that distinguishing presence versus absence of the cause in diagnostic information requires an extra cognitive effort. Thus, simply processing the complex stimuli used by Blanco et al. (2020) would take more cognitive effort and make it difficult for participants to distinguish the presence versus absence of the cause as a specific diagnostic variable. In Study 1, very simple cues were employed, which would reduce the effort required to process this information. In this study, we attempted to examine this possibility by using a more complex, although still discrete, set of cues. Specifically, we used six state indicators, which allowed for seven different combinations (three with more positive than negative cues, one with equal number of both types, three with more negative than positive cues). This allows examination of the effects of using more complex cues, and in addition provides additional support for the basic findings of Blanco et al. (2020).

### Method

#### Participants

In all, 250 participants were recruited from the online platform Prolific (165 females, 87 males, mean age = 40.7). The same criterion as in Study 1 was used to determine the stopping rule (i.e., 150 participants classified as Statistical or Counterexample).

### Material

#### Strategy Assessment Task

To evaluate the Strategy, we used the same procedure as in Study 1.

#### Contingency Learning Task

We replicated the same procedure as in Study 1. Participants were still asked to imagine that they were a psychologist and that they wanted to evaluate the efficacy of a protocol to cure “Hylophobia.” However, we added two new dichotomous indicators of state (i.e., indicators E and F) to increase the number of combinations with both positive and negative ratios of evidence. Thus, during the learning phase, participants were exposed to seven different combinations, synthesised in Table 2. For each trial, participants were informed whether the patient received the treatment or not, and based on the indicators, they were asked to evaluate whether the patient was cured or not (Dichotomous judgement Yes / No). For all trials, the position of faces was counterbalanced. Before the learning phase, participants were given two examples: one with all smiling faces and the other with all sad faces. We kept the number of trials equivalent to Study 1 (i.e., 52 trials) to control for the potential effect of the number of trials on judgement. There were 20 trials with an equal amount of positive and negative cues (i.e., completely ambiguous trials), 16 trials with a positive ratio (i.e., the number of smiling faces was superior to the number of sad faces), and 16 trials with a negative ratio. Because we kept the number of trial equivalent to Study 1, it was not possible to have an equal number of each combination for the positive and negative ratio trials. Thus, 5:1 and 1:5 ratios were presented four times, while the other ratios were presented six times. The rate at which the treatment was given was the same across trials (see Table 2). Contingency computed with trials with more positive cues than negative cues as patient cured and trials with more negative cues than positive as patient not cured was null ( $\Delta_{p}=0)$ .

**Table 2.** Number of trials for each type of ratio of evidence.

	Positive ratio			Equal ratio	Negative ratio		
Ratio (smiling faces:sad faces)	6:0	5:1	4:2	3:3	2:4	1:5	0:6
Cause present	3	2	3	10	3	2	3
Cause absent	3	2	3	10	3	2	3

As in Study 1, at the end of the learning phase participants were asked to evaluate the efficacy of the protocol using a scale from 0 (“Definitely No”) to 100 (“Definitely Yes”).

#### Procedure

Participants were presented with both the Strategy Assessment Task and the Contingency Learning Task. The order of presentation of the tasks was counterbalanced. Furthermore, in the Contingency Task the order of the trials was randomised.

### Results

#### Categorisation of trials

First, we examined whether the rate at which participants categorised patients as cured or not varied according to the ratio of evidence (i.e., number of green faces) and the presence or absence of the putative cause. We computed a Generalised Mixed Model (using lme4 package for R) using a binary logistic regression with the categorisation of each trial (0 = effect absent, 1 = effect present) as the dependent variable and number of green Face, Cause (Absent, present), and the interaction between these terms as independent variable. We used Participant and Item as random effects to account for the repeated measures (the model was Categorisation ~ Faces × Cause (1|Id) + (1|Item)). This gave a significant main effect for Faces, $\chi^{2}$ (1, *N* = 252) = 395.64, *p* < .001. There was no other significant effect. As in Study 1, the number of green faces and the level of categorisation of trials as patient cured followed a positive linear relationship.

We then examined results on the strategy assessment task. We used the same criteria as in Study 1. This gave 59 counterexamples, 111 statistical, 31 Intermediate reasoners, and 51 Others. As in Study 1, we report only the direct comparisons between Statistical and Counterexample reasoners. The results for the Intermediate reasoners and Other participants are presented in Supplementary Material 2.

We computed a Generalised Mixed Model (lme4 package for R, emmeans for contrast analysis with Holm’s correction) using a binary logistic regression with the Categorisation of each trial (0 = patient not cured, 1 = patient cured) as the dependent variable and Strategy (Counterexample, Statistical, Intermediate, Other), Cause (Absent, Present), and number of green Faces along with their interaction as independent variables. We included Participant as a random effect to account for the repeated measures (the model was Categorisation ~ Strategy × Ratio + (1|Id) + (1|Item)).

As in Study 1, this gave significant main effects for Strategy, $\chi^{2}$ (3, *N* = 252) = 25.52, *p* < .001, and Faces, $\chi^{2}$ (1, *N* = 252) = 433.14, *p* < .001. However, unlike Study 1, the main effect of Cause was not significant, $\chi^{2}$ (1, *N* = 252) = 3.59, *p* = .06. We also found the same significant effects for the Strategy × Faces interaction, $\chi^{2}$ (3, *N* = 252) = 69.85, *p* < .001, and the three-way interaction Strategy × Cause × Faces, $\chi^{2}$ (3, *N* = 252) = 11.50, *p* = .009). The Strategy × Cause interaction was not significant, $\chi^{2}$ (3, *N* = 252) = 0.2, *p* = .9.

The main effect of Faces showed a positive linear relationship between the number of faces and the level of categorisation of trials as patient cured. The main effect of Strategy showed that Statistical reasoners (*EMM* = 0.07, *SE* = 0.02) categorised significantly more often trials as patient cured than Counterexample reasoners (*EMM* = 0.009, *SE* = 0.004).

Analysis of the Strategy × Faces interaction showed the same pattern as found in Study 1, that is, the correlation between Faces and Categorisation of trials as patient cured was stronger for Counterexample reasoners (*b* = 3.40, *SE* = 0.20) than for Statistical reasoners (*b* = 2.91, *SE* = 0.15).

Finally, the three-way interaction showed the same pattern of results as in Study 1, that is, for both strategies and when the cause was both present and absent, the number of green Faces predicted the level of categorisation of trials as patient cured. The correlation between the number of green faces and the categorisation of trials as patient cured was stronger for Counterexample (Cause Absent: *b* *=* 3.07, *SE* = 0.23, Cause Present: *b* = 3.73, *SE* = 0.27) than Statistical reasoners (Cause Absent: *b* = 2.66, *SE* *=* 0.18, Cause Present: *b* = 3.15, *SE* *=* 0.20) when the cause was both absent and present. Also, for both strategies, the correlation was stronger when the cause was present than when it was absent.

**Figure 5.**
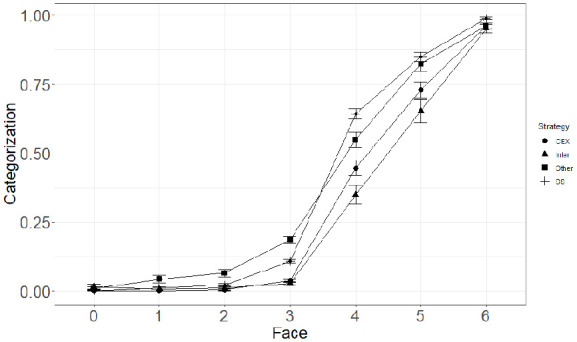
Mean levels of categorisation as a function of the number of green faces and the reasoning strategy.

As in Study 1, we computed pairwise comparisons between Strategies for each level of Faces (Wilcoxon test, Holm’s adjustment—see Figure 5). The same pattern of results as in Study 1 was observed for non-ambiguous trials, that is, trials with zero and six green faces, and Counterexample and Statistical reasoners classified trials similarly both when the putative cause was present and absent.

As in Study 1, the same pattern of categorisation of trials was observed for trials with less green than red faces and truly ambiguous trials. Trials with one and two green faces were categorised as patient cured similarly between Counterexample and Statistical reasoners, both when the putative cause was present and absent. For truly ambiguous trials, both when the putative cause was present and absent, Counterexample reasoners classified trials as patient cured less often than Statistical reasoners.

For trials with four green faces, Counterexample classified less often trials as patient cured, both when the putative cause was present and absent. Finally, for the remaining trials (i.e., five green face trials), categorisation of trials was similar between Counterexample and Statistical reasoners both when the putative cause was present and absent.

#### Effect of categorisation on judgements

As in Study 1, we first computed the proportion of trials categorised as patient cured (subjective P(O)). Then, we categorised each event using the same typology as in Study 1, that is, treatment was given and the patient was considered cured was a Type a event, and so on. This was used to calculate the subjective contingency (i.e., ∆*_p_*).

**Figure 6.**
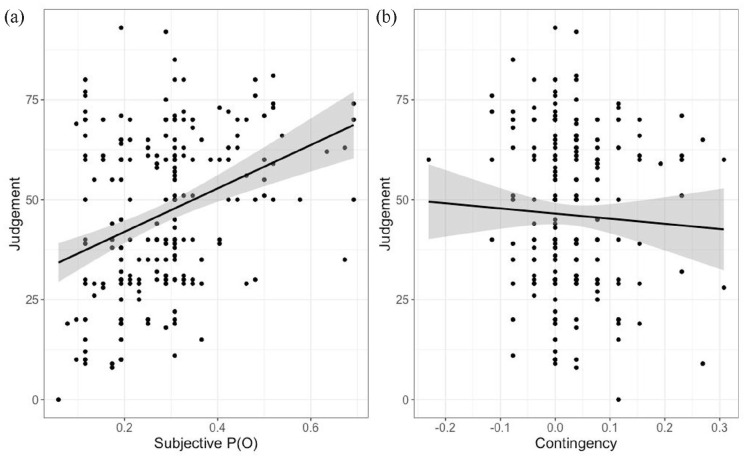
Scatterplot of judgements as a function of subjective P(O) (a) and contingency (b) in Study1.

We then computed a linear regression with Judgement of efficacy as the dependent variable and subjective P(O) and Contingency as independent variables. This gave a significant effect of subjective P(O), *b* = 54.04, *t*(249) = 5.52, *p* < .001, but not of contingency, *b* = −9.46, *t*(249) = −0.56, *p* = .6 (see Figure 6).

We then examined whether strategy use influenced the relationship between categorisation of trials and judgement. First, we computed a linear regression with judgement as the dependent variable and Strategy and Contingency as independent variables. This showed a significant difference between Statistical and Counterexample reasoners, *b* = 8.22, *t*(247) = 2.52, *p* = .012. No other results were significant. We examined the effect of Strategy by conducting post hoc analysis (Tukey’s correction, *p* = .05). Mean judgements of Statistical reasoners (*M* = 48.7, *SE* = 19.4) were significantly higher than Counterexample reasoners (*M* = 40.5, *SE* = 21.3).

**Figure 7.**
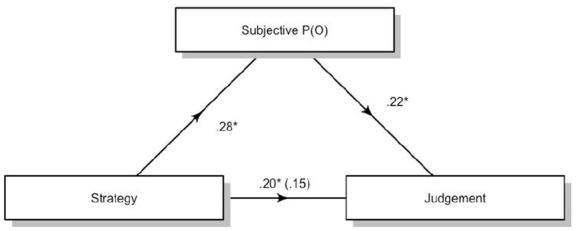
Standardised regression coefficients for the relationship between Strategy (Counterexample and Statistical reasoners only) and Judgement as mediated by Subjective P(O) in Study 2. The coefficient between Strategy and Judgement while controlling for Subjective P(O) is in parentheses. *p < .05.

Finally, we conducted a mediation analysis (bootstrapping 5,000 samples) with Judgement as the dependent variable, Strategy as the independent variable, and subjective P(O) as the mediator. This gave a significant effect for the indirect effect of subjective P(O), *b* = 2.1, 95% CI = [0.04, 4.15]. The residual indirect effect was significant, *b* = 8.19, 95% CI = [1.86, 14.53] (Figure 7).

## General discussion

In these studies, we examined whether the dual strategy framework could explain individual differences in the categorisation of ambiguous trials in contingency learning tasks. The dual strategy model proposes a difference between two kinds of reasoning strategy, Counterexample and Statistical. Counterexample reasoners use a more focused representation of information that is treated by working memory–intensive processes, while Statistical reasoners use a more intuitive form of information activation and treatment. Previous results (Béghin et al., 2021; Béghin & Markovits, 2022) have indeed shown that reasoning strategy predicts clear differences in the way that contingency information is processed. However, these studies were conducted in classical contingency learning paradigms (i.e., using binary cause and effect). In the present studies, we attempted to extend this relation to the specific case of the interpretation of ambiguous cues to outcomes.

In many real-life diagnostic situations, people must infer the efficacy of a putative treatment based on a variety of diagnostic cues. Blanco et al. (2020) presented people with a contingency learning task where the real contingency was 0 (i.e., there was no objective causal effect of a hypothetical treatment on a hypothetical illness). Participants were given complex indicators of patient outcomes (2,500 light or dark squares) and asked to classify whether these are indicating either that the patient was cured or not. They found that the extent to which participants interpret objectively ambiguous states (equal numbers of light and dark cells) as indicating that the patient was cured determined their final judgements of the efficacy of the treatment. To broaden these results, we conducted two null contingency learning tasks where participants were asked to evaluate the efficacy of a treatment for a mental disorder. In each trial, participants had to interpret whether the patient was cured or not using a relatively small number of dichotomous cues to patient state (i.e., four indicators in Study 1; six indicators in Study 2). Our basic prediction here was that even with a smaller number of cues to patient state, there would be a clear relation between the tendency to interpret ambiguous cues as indicating that the patient was cured and final judgements of efficacy.

Now, previous studies have shown a clear relationship between strategy use and contingency judgements (Béghin et al., 2021; Béghin & Markovits, 2022). These show that, given the same profile of contingency information, Counterexample reasoners generate lower estimates of causal efficacy than Statistical reasoners. However, the present studies examine another unrelated effect, the tendency to interpret ambiguous cues to patient state as indicating a state of wellness. One of the key differences between Counterexample and Statistical reasoners is that, given both positive and negative forms of information, the latter give more weight to positive forms of information (e.g., Markovits et al., 2018). As ambiguous states are by definition composed of both positive and negative information, we hypothesised that Statistical reasoners would be more prone to give more weight to positive cues relative to Counterexample reasoners and thus produce higher levels of evaluations of patient state as indicating that the patient was cured, and correspondingly higher levels of treatment efficacy than Counterexample reasoners.

These basic predictions were confirmed in both studies. First, as hypothesised, Statistical reasoners showed a greater tendency to categorise trials as indicating that the patient was cured than did Counterexample reasoners. This difference was specifically true for truly ambiguous trials, that is, trials with the same number of positive and negative evidence (both when the putative cause was present and absent). Also, significant differences were found for trials with one more positive than negative cue, when the putative cause was absent in Study 1, and both when the putative cause was present and absent in Study 2.

This is consistent with the idea that Counterexample reasoners attended more to both the positive and negative evidence, while Statistical reasoners put more emphasis on the former. In addition, mediation analyses indicated that final judgements of efficacy were related to the tendency for Statistical reasoners to interpret ambiguous cues more positively and for these reasoners to generate higher judgements with the same information. This adds more weight to the usefulness of the Counterexample/Statistical distinction as an important component to our understanding of individual differences in contingency learning.

In both studies, we concentrated on the Counterexample/Statistical distinction. However, we also included analyses with both the Intermediate reasoners and Other participants. As suggested above, Intermediate reasoners are identified as generally using a Counterexample strategy with occasional lapses into a Statistical strategy. Results of both studies indicated that Intermediate reasoners had levels of categorisation of trials that were situated between those of Counterexample and Statistical reasoners, but closer to those of the former, which is quite consistent with our analysis of Intermediate reasoners.

On the other hand, the nature of the Other category is less clear. Previous studies (Thompson & Markovits, 2021) showed that Other participants had lower cognitive abilities and responded less consistently to the diagnostic problems. This category of participants tends to produce results lower than those of Statistical reasoners. In the present studies, Other participants showed levels of categorisation of trials that were similar to those of Statistical reasoners. It may be that these participants find categorisation less difficult than the reasoning tasks used in the diagnostic questionnaire, thus underestimating their difficulties. Nonetheless, it indicates the usefulness of additional studies that examine more specifically how these Other participants react to different kinds of judgements.

Second, the results of these studies extend the findings of Blanco et al. (2020) by showing that the interpretation of ambiguous states is uniquely related to final judgements of ambiguity even when the number of cues to patient state is limited. In both the relevant studies of Blanco et al. (2020) and the present ones, the objective contingency between the proposed treatment and outcomes was null. Even when contingency information is updated to include the way that outcomes were categorised, there was no relation between this and real contingency information, even when this was adjusted for the way that ambiguous cues were evaluated. Rather, people’s judgements of efficacy were solely related to the extent to which they tended to evaluate patient states having both positive and negative information as indicating a state of wellness.

There was, however, one interesting divergence between the Blanco et al. (2020) results and those of the present studies. In Study 1, there was a clear effect of the presence or absence of putative treatment, with the former generating a stronger tendency to rate ambiguous states as indicating patient cured. However, this effect was not present in Study 2, nor in the Blanco et al. (2020) study using a null contingency task. It should be noted that Blanco et al. (2020) replicated their study using a positive contingency learning task ( $\Delta_{p}=0.86)$ . Under positive contingency, participants tended to categorise ambiguous trials as patient cured more often when the putative cause was present than when it was absent. This tendency appeared after being exposed to trials suggesting the existence of a positive contingency, which suggests some kind of confirmation bias where participants end up categorising ambiguous trials consistently with the overall contingency. While speculative, it is useful to note that Study 1 presented a minimal number of cues to patient state, while both Study 2 and the Blanco et al. (2020) study used more complex cues. It is possible that when the contingency is not extreme (i.e., neither high nor low) the cognitive load required to process more complex representations of patient state might interfere with the concurrent processing of presence/absence of the treatment. This is, in turn, consistent with the general finding that judgements of efficacy are not related to contingency information, but only to the tendency to rate ambiguous states as patient cured, at least in a null contingency situation.

One final point should be addressed here. Although the dual strategy model supposes that there is a qualitative difference between Counterexample and Statistical reasoners, the present results could also be interpreted within a signal detection framework. Simply put, it is possible that the former have a more conservative threshold for making any kind of decision, which would explain the key differences observed in this study. While this interpretation has been addressed previously (see Thompson & Markovits, 2021), it is worth making the following point. Counterexample reasoners are those who reject all 10 of the inferences in the Strategy diagnostic instrument. Statistical reasoners, thus, reject fewer than this number. However, if the number of rejected inferences is indeed a unique marker of individual differences in people’s decision thresholds, then this should be as important a factor within the category of Statistical reasoners. Indeed, examination of the results of the two studies found that the total number of rejected inferences for participants categorised as Statistical reasoners varied between 8 and 2, which would imply that there should be large corresponding differences in thresholds within this category. However, the correlation between the number of rejected inferences on the diagnostic instrument and the proportion of trials categorised as patient cured was not significant, either in Study 1, *r*(102) = −.041, *p* = .680, or in Study 2, *r*(109) = −.10, *p* = .30. Nonetheless, it remains possible that some form of signal detection model might underlie the basic distinction made by the dual strategy model. Further research would be required to examine this possibility.

One limitation to this study is that participants were asked to make a forced choice between the patient being cured or not for each trial. It would certainly be interesting to examine performance if a third, neutral, option was added. However, the forced choice could not in itself account for the pattern of positive interpretations of ambiguous states. In addition, it could be argued that in a clinical context, patients are not evaluated as being in a neutral state. They can be seen as cured, ill, or improving. However, improving (i.e., having fewer symptoms), is still being ill and requiring more treatment. In addition, some studies have suggested that participants might dichotomise cues even when they were not forced to, depending on their basic causal hypotheses (e.g., Marsh & Ahn, 2009). A second limitation is the use of a mental illness as the target condition. Mental illnesses might not be considered to be as amenable to a cure as a more well-defined physical condition. Thus, while these results are a generalisation of those of Blanco et al. (2020), the overall parameters, such as the overall rate of interpreting ambiguous cues, might be affected by this choice. Nonetheless, the correlations between the way that ambiguous cues are interpreted and judgements of efficacy and that between strategy use and these two parameters are not affected by choice of illness. Another possible limitation concerns the question asked to participants to judge the efficacy of the treatment. Participants were asked to what extent they thought the protocol helped reduce the symptoms of Hylophobia, which could have led to higher judgements than if they were asked to rate the extent to which they thought the protocol cured the disorder.

In sum, we report two main findings here. First, there is a clear relation between the tendency to categorise ambiguous trials as patient cured and subsequent judgements of the efficacy of the treatment, even when there is no objective evidence for a causal relation between treatment and illness. Second, the dual strategy model provides a framework to understand individual differences in the categorisation of trials in contingency learning. While participants tended to be generally conservative in their interpretation of ambiguous cues, when there were equal or greater numbers of positive cues, Statistical reasoners tended to conclude more often than Counterexample reasoners that these cues indicated that the patient was cured. This is consistent with the hypothesis that Statistical reasoners give more weight to positive information than Counterexample reasoners. Overall, these results show that the effect of reasoning strategy on judgements of efficacy is a combination of two tendencies: (1) the tendency of Statistical reasoners to evaluate ambiguous cues as indicating patient cured more often than Counterexample reasoners and (2) the tendency of Statistical reasoners to evaluate the same contingency information as indicating a higher level of treatment efficacy compared with Counterexample reasoners. This provides additional support to the idea that the dual strategy model provides a useful framework to study individual differences in contingency learning (Béghin et al., 2021; Béghin & Markovits, 2022).

## Supplemental Material

sj-docx-1-qjp-10.1177_17470218231155897 – Supplemental material for Interpretation of ambiguous trials along with reasoning strategy is related to causal judgements in zero-contingency learning Supplemental material, sj-docx-1-qjp-10.1177_17470218231155897 for Interpretation of ambiguous trials along with reasoning strategy is related to causal judgements in zero-contingency learning by Gaëtan Béghin and Henry Markovits in Quarterly Journal of Experimental Psychology

## Notes

1 However, we have also analysed the Intermediate and Other categories, analyses which are given more completely in Supplementary Material 2.
