# Supplementary material for: Interpretation of ambiguous trials along with reasoning strategy is related to causal judgements in zero-contingency learning
Source: Q J Exp Psychol (Hove). 2023 Feb 24;76(12):2704–17. doi: 10.1177/17470218231155897 (PMC10663643; doi:10.1177/17470218231155897)
Supplement: sj-docx-1-qjp-10.1177_17470218231155897 – Supplemental material for Interpretation of ambiguous trials along with reasoning strategy is related to causal judgements in zero-contingency learning [file sj-docx-1-qjp-10.1177_17470218231155897.docx]

Supplementary Material for:

**Interpretation of ambiguous trials along with reasoning strategy are related to causal judgments in zero-contingency learning**

Gaëtan Béghin

Henry Markovits

Université du Québec à Montréal

Corresponding author: Henry Markovits, [henrymarkovits@gmail.com](mailto:henrymarkovits@gmail.com)

Author emails: Gaëtan Béghin, [beghingaetan@gmail.com](mailto:beghingaetan@gmail.com)

**Supplementary Material 1**

**Contingency task instructions**

*Imagine that you are a psychologist specialized in a new rare mental health disorder called Hylophobia. You recently developed a protocol to treat this condition. The protocol consist of 12 individual sessions of therapy, conducted by a psychologist, at a rate of one session per week. To test for the efficacy of your protocol, you conducted an experiment.
In your experiment, all participants were previously diagnosed with Hylophobia. From the 52 participants that you recruited, some were given your treatment protocol, the others were not.*

***For all of these participants, you not only created the therapy, but you personally did the therapy sessions.***

*At the end of the 12 weeks, you measured 4 indicators of symptoms that allow you to know to what extent the patient still has the disorder.
You will be presented with the results of your experiment. For each participant you will know if they received the treatment as well as their results on each indicator. To help you with the interpretation of the indicators, you know that when the result of a specific indicator is good, a green smiling face is presented. Reciprocally, when the result is not good, a red sad face is presented.
Based on the 4 indicators, you will be asked to judge if the participant recovered or not. At the end of the presentation of all cases, you will be asked to judge, based on the results, to what extent you think that the protocol reduced Hylophobia symptoms in participants.
Before starting, you will be presented with two examples. 
Click on the arrow to receive the examples.*

**Contingency learning task examples**

Example 1:

***“This is an example. For each trial, the patient’s ID is exposed as well as if he received the protocol. In this example, the patient received the treatment and all indicators are a green smiling face, indicating that they are good.***
***You have to decide if the patient is cured or not.***
***Click on one of the possibility to see another example.***

*Patient XPEX1****The protocol was administered***

| *Indicator A* | *Indicator B* | *Indicator C* | *Indicator D* |
| --- | --- | --- | --- |
| *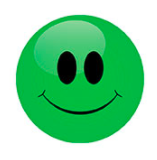* | *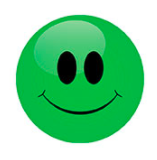* | *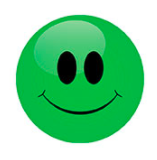* | *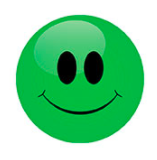* |

- The patient is cured
- The patient is not cured”

Example 2:

***This is the same example. However, now all indicators are a red sad face, indicating that all indicators are bad.***

***You have to decide if patient is cured or not.***

***Patient XPEX1
The protocol was administered***

| ***Indicator A*** | ***Indicator B*** | ***Indicator C*** | ***Indicator D*** |
| --- | --- | --- | --- |
| *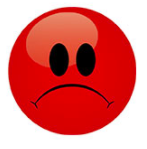* | *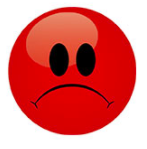* | *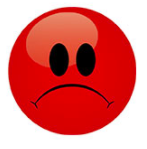* | *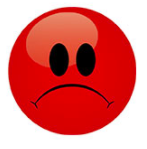* |

**Strategy assessment task**

**Instructions**

*For this task, imagine that scientists discovered a new inhabited planet, called Planet Kronus.*

*A team of scientists was then sent to this planet. These scientists discovered some things that do not exist on Planet Earth. In the following pages, you will see a description of their discoveries. Read these carefully, because they give important information about the discoveries.*

*For each discovery, you will be given a rule that has been confirmed by scientists, which you must consider to be true. It is very important that you suppose that each rule presented by the scientists is always true when responding to the following questions. Following each rule, an observation and a conclusion will be presented.

You must indicate whether or not the conclusion can be logically drawn from the presented information.*

*Please take your time to answer the questions.*

In the following MP-X correspond to the Modus Ponens items. The AC_10 items are Affirmation of the consequent with the associated frequencies suggesting a high probability of P being true (around 90%), whereas the AC_50 are also Affirmation of the consequent but the frequencies suggest a low probability of P being true (around 50%)

**Item 1 (MP1)**

A team of meteorologists watching the local climate of the planet Kronus noted an interesting phenomenon. They noted that on Kronus: **If it thardonnes, then the soil will become sticky**

On the last 1000 times it thardonned, meteorologists made the following observations:

**1000 times it thardonned and the soil became sticky.**

**0 times it has not thardonned and the soil became sticky.**

From this information, John reasoned as follows:

If it thardonnes, then the soil will become sticky

Observation : **It thardonnes**.

Conclusion :**The soil will become sticky.**

Indicate whether the conclusion of John can be drawn logically or not from the information provided.

- Yes
- No

**Item 2 (AC_10_1)**

By exploring a cave of Kronus, geologists have discovered a variety of very special stone, trolyte. Following a series of observations, they argue that on Kronus: **If trolyte is heated, then it will release philoben gas.**

On the last 1000 times they observed trolytes, geologists have made the following observations:

**900 trolytes were heated and release philoben gas.**

**100 trolytes were not heated and released philoben gas.**

From this information, Mary reasoned as follows:

If trolyte is heated, then it will release philoben gas.

Observation: **A trolyte released philoben gas.**

Conclusion: **The trolyte was heated.**

Indicate whether the conclusion of Mary can be drawn logically or not from the information provided.

- Yes
- No

**Item 3 (AC_50_1)**

By studying the unique wildlife of Kronus, biologists have made a discovery about the birds. They state that: **If a bird has water in its beak, then the color of its plumage will change.**

Of the 1,000 birds they examined recently, scientists have made the following observations:

**500 birds had water in their beak and the colour of their plumage has changed.**

**500 birds have not had water in their beak and the colour of their plumage has changed.**

From this information, James reasoned as follows:

If a bird has water in its beak, then the color of its plumage will change.

Observation: **The colour of the plumage of a bird has changed.**

Conclusion: **The bird has water in its beak.**

Indicate whether the conclusion of James can be drawn logically or not from the information provided.

- Yes
- No

**Item 4 (MP2)**

A group of botanists studying the flora has discovered a unique property of plants growing in the soil of Kronus. According to botanists, on Kronus: **If X45 fertilizer is given to a plant, then the plant will become phosphorescent.**

Over the last 1,000 times they observed the plants, the scientists made the following observations:

**1,000 plants were given X45 fertilizer and became phosphorescent.**

**0 plants were not given X45 fertilizer and became phosphorescent.**

From this information, Linda reasoned as follows:

If X45 fertilizer is given to a plant, then the plant will become phosphorescent.

Observation: **A plant is given X45 fertilizer.**

Conclusion: **The plant will become phosphorescent.**

Indicate whether the conclusion of Linda can be drawn logically or not from the information provided.

- Yes
- No

**Item 5 (AC_10_2)**

A team of chemists is mixing different substances unique to the planet Kronus. Following a series of observations, they affirm that on Kronus: **If fannar is mixed with water, then it will become yellow.**

On the last 1,000 observations made, the chemists noted that:

**910 times fannar has been mixed with water and became yellow.**

**90 times the fannar has not been mixed with water and became yellow.**

From this information, Robert reasoned as follows:

If fannar is mixed with water, then it will become yellow.

Observation: **A plant is given X45 fertilizer.**

Conclusion: **The plant will become phosphorescent.**

Indicate whether the conclusion of Robert can be drawn logically or not from the information provided.

- Yes
- No

**Item 6 (AC_50_2)**

A team of meteorologists watching the local climate of the planet Kronus noted an interesting phenomenon. They noted that on Kronus: **If the sun shines, then the ground will become green.**

On the last 1000 times when they observed the ground, meteorologists made the following observations:

**505 times the sun was shining and the ground became green.**

**495 times the sun did not shine and the ground became green.**

From this information, Jennifer reasoned as follows:

If the sun shines, then the ground will become green.

Observation: **The ground became green.**

Conclusion: **The sun was shining.**

Indicate whether the conclusion of Jennifer can be drawn logically or not from the information provided.

- Yes
- No

**Item 7 (MP3)**

Chemists working on Kronus found that the water was very special. Following a series of observations, they argue that on Kronus: **If we boil water, then the water will become red.**

On the last 1,000 experiments they made, chemists have made the following observations:

**1000 times, boiling water became red.**

**0 times, water was not boiled and became red.**

From this information, William reasoned as follows:

If we boil water, then the water will become red.

Observation: **Water is boiled.**

Conclusion: **The water will become red.**

Indicate whether the conclusion of William can be drawn logically or not from the information provided.

- Yes
- No

**Item 8 (AC_10_3)**

By exploring a cave of Kronus geologists have discovered a particular property of rocks. Following a series of observations, they argue that on Kronus: **If a rock is made wet, then it will change color.**

Over the last 1,000 times they observed rocks, geologists have made the following observations:

**920 rocks were made wet and have changed color**

**80 rocks were not made wet and have changed color.**

From this information, Susan reasoned as follows:

If a rock is made wet, then it will change color.

Observation: **A rock changes colour.**

Conclusion: **The rock was made wet.**

Indicate whether the conclusion of Susan can be drawn logically or not from the information provided.

- Yes
- No

**Item 9 (AC_50_3)**

By studying the unique wildlife of Kronus, biologists have made a discovery about cats on Kronus. They state that: **If you feed a cat, then its eyes turn red.**

On the 1000 cats they examined, scientists have made the following observations:

**510 cats have been fed and their eyes have turned red.**

**490 cats have not been fed and their eyes have turned red.**

From this information, David reasoned as follows:

If you feed a cat, then its eyes turn red.

Observation: **A cat has red eyes.**

Conclusion: **The cat was fed.**

Indicate whether the conclusion of Susan can be drawn logically or not from the information provided.

- Yes
- No

**Item 10 (AC_10_4)**

A group of botanists studying the flora discovered another unique property of plants growing in the soil of Kronus. According to botanists, on Kronus: **If a plant is watered, then the plant will become orange.**

Over the last 1,000 times they observed the plants, the scientists made the following observations:

**905 plants were watered and became orange.**

**95 plants were not watered and became orange.**

From this information, Lisa reasoned as follows:

If a plant is watered, then the plant will become orange.

Observation: **A plant became orange.**

Conclusion: **The plant was watered.**

Indicate whether the conclusion of Lisa can be drawn logically or not from the information provided.

- Yes
- No

**Item 11 (AC_50_4)**

A team of biologists on Kronus has discovered a very special animal, kikina. Following a series of observations on the animal, they argue that on Kronus: **If a kikina eats meat, then it will shrink.**

On the last 1,000 experiments they made, biologists have made the following observations:

**520 kikinas ate meat and shrunk.**

**480 kikinas did not eat meat and shrunk.**

From this information, Charles reasoned as follows:

If a kikina eats meat, then it will shrink.

Observation: **A kikina shrunk.**

Conclusion: **The kikina ate meat.**

Indicate whether the conclusion of Charles can be drawn logically or not from the information provided.

- Yes
- No

**Items 12 (AC_10_5)**

A group of botanists studying the different trees on Kronus discovered that they have a unique property. According to botanists, on Kronus: **If you burn a tree, then purple smoke will be produced.**

**915 times trees have been burned and purple smoke was produced.**

**85 times trees have not been burned and purple smoke was produced.**

From this information, Nancy reasoned as follows:

If you burn a tree, then purple smoke will be produced.

Observation: **Purple smoke was produced.**

Conclusion: **A tree was burned.**

Indicate whether the conclusion of Nancy can be drawn logically or not from the information provided.

- Yes
- No

**Item 13 (AC_50_5)**

A team of biologists has discovered on Kronus an animal having unique properties, the tritana. Following a series of observations, they argue that on Kronus: **If a tritana is sprayed with alcohol, then it will emit a sharp sound.**

On the last 1,000 experiments they made, geologists have made the following observations:

**515 tritana have been sprayed with alcohol and have emitted a sharp sound.**

**485 tritana have not been sprayed with alcohol and have emitted a sharp sound.**

From this information, Thomas reasoned as follows:

If a tritana is sprayed with alcohol, then it will emit a sharp sound.

Observation: **A tritana emits a sharp sound.**

Conclusion: **The tritana was sprayed with alcohol.**

Indicate whether the conclusion of Thomas can be drawn logically or not from the information provided.

- Yes
- No

**To calculate reasoning Strategy please use the instructions available in the key data at:** https://osf.io/cw2a8/?view_only=4baf1f3a486b43758a39bc36da064c50

**Supplementary Material 2**

In this section of the Appendix, we report the full contrasts from the analyses with the full range of reasoning strategies.

**Study 1**

**Categorization of trials**

Results from the Generalized Mixed Model using Categorization as the dependent variable, Strategy, Cause, and Faces along the interactions between these terms as independent variables showed a main effect for Face, Cause and Strategy. The two-ways interactions Strategy X Cause, Strategy X Face were also significant. Finally, the three-way interaction Face X Cause X Strategy was significant. Results from the contrast analyses for the main effects of Strategy showed that Intermediate reasoners (*EMM* = 0.03, *SE* = 0.01) level of categorization of trials as patient cured was similar to the one of Counterexample reasoners (*EMM* = 0.02, *SE* = 0.006) but it was significantly lower than Statistical reasoners (*EMM* = 0.14, *SE* = 0.03) and Other participants (*EMM* = 0.20, *SE* = 0.05) level of categorization. Level of categorization of trials as patient cured was similar between Statistical reasoners and Other reasoners.

Analysis of the Strategy X Faces interaction showed that for all strategies, the number of green faces significantly predicted the level of categorization of trials as patient cured. The correlation between Categorization and number of Faces was similar between Counterexample (*b* = 4.11, *SE =* 0.25) and Intermediate reasoners (*b* = 3.59, *SE* = 0.25) but were significantly higher than for Statistical reasoners (*b* = 2.97, *SE* = 0.13) and Other reasoners (*b* = 2.17, *SE* = 0.13). The correlation between Categorization and number of green Faces was similar between Statistical reasoners and Other participants. Analysis of the Strategy X Cause interaction showed the same pattern both when the putative cause was present and absent. Categorization levels of trials as patient cured were similar between Counterexample (Cause Absent: *EMM*  = 0.01, *SE*  = 0.004, Cause Present : *EMM* = 0.03, *SE* = 0.01) and Intermediate reasoners (Cause Absent: *EMM* = 0.02, *SE* =0.009, Cause Present: *EMM* = 0.04, *SE =* 0.02) but were lower than Statistical reasoners (Cause Present: *EMM* = 0.12, *SE*  = 0.03, Cause Present : *EMM* = 0.17, *SE* = 0.04) and Other participants (Cause Present: *EMM =*0.16, *SE* = 0.04, Cause Absent : *EMM*  = 0.12, *SE =* 0.03). Finally, analysis of the three-way interaction Cause X Faces X Strategy showed that the correlation between Categorization of trials and number of green Faces when the cause was absent was similar between Counterexample reasoners (*b* = 3.38, *SE* = 0.26) and Intermediate reasoners (*b* = 3.48, *SE =* 0.30), but were higher than Statistical (*b* = 2.56, *SE* = 0.18) and Other (*b =* 2.06, *SE* = 0.17) reasoners. When the treatment was present, the correlation between Categorization of trials and the number of Faces was higher for Counterexample (*b* = 4.83, *SE* = 0.35) than for Intermediate (*b* = 3.70, *SE* = 0.31), Statistical (*b* = 3.38, *SE* = 0.18) and Other (*b =* 2.27, *SE =* 0.17*)* reasoners. They were similar between Intermediate and Statistical reasoners, but higher than Other participants. For both Statistical and Counterexample reasoners the correlation was higher when the putative cause was present than when it was absent, whereas for Intermediate reasoners and Other participants correlations were similar – see Figure A1.

We then computed pairwise comparisons between Strategies for each level of Faces and Cause (Wilcoxon, Holm’s correction). For trials with 0 green faces, categorization of trials as patient cured was similar both when the putative cause was present and absent. The same pattern was found for trials with 1 green face when the putative cause was absent. For trials with 1 green face and the putative cause, categorization levels of trials as patient cured were significantly higher for Other participants than for Counterexample reasoners. Other comparisons were not significant. For truly ambiguous trials when the putative cause was absent, Counterexample and Intermediate reasoners had similar levels of categorization of trials as patient cured. Counterexample reasoners classified less often patient as cured than both Other participants and Statistical reasoners. Also, Intermediate reasoners classified less often patient as cured than Other participants. However, classification was similar between Statistical reasoners and Intermediate reasoners, and Statistical reasoners and Other participants.

For trials with 3 green faces when the putative cause was absent, Intermediate, Counterexample reasoners and Other participants had similar levels of trials categorized as patient cured. Also, both Counterexample and Intermediate reasoners categorized less often as patient cured than Statistical reasoners. Other participants and Statistical reasoners had similar levels of categorization. Finally, for trials with four green faces, levels of categorization were similar between strategies both when the putative cause was present and absent.

Table A.1 Mean levels of categorization of trials as patient cured as a function the Face, Cause and Strategy in Study 1

| Ratio (smiling faces: sad faces) | Cause | CEX | Inter | Other | SS |
| --- | --- | --- | --- | --- | --- |
| 0:4 | P | 0.005 (0.034) | 0.015 (0.060) | 0.01 (0.078) | 0.019 (0.096) |
|  | A | 0.00 (0.00) | 0.0076 (0.043) | 0.046 (0.16) | 0.005 (0.035) |
| 1:3 | P | 0.009 (0.048) | 0.0074 (0.043) | 0.063 (0.15) | 0.033 (0.11) |
|  | A | 0.00 (0.00) | 0.030 (0.082) | 0.054 (0.15) | 0.036 (0.12) |
| 2:2 | P | 0.028 (0.14) | 0.071 (0.016) | 0.19 (0.26) | 0.11 (0.19) |
|  | A | 0.085 (0.27) | 0.12 (0.23) | 0.26 (0.31) | 0.17 (0.25) |
| 3:1 | P | 0.415 (0.46) | 0.42 (0.47) | 0.60 (0.40) | 0.712 (0.38) |
|  | A | 0.60 (0.47) | 0.48 (0.48) | 0.63 (0.41) | 0.77 (0.36) |
| 4:0 | P | 0.82 (0.33) | 0.98 (0.10) | 0.90 (0.26) | 0.92 (0.24) |
|  | A | 1.00 (0.00) | 0.98 (0.07) | 0.98 (0.11) | 0.99 (0.05) |


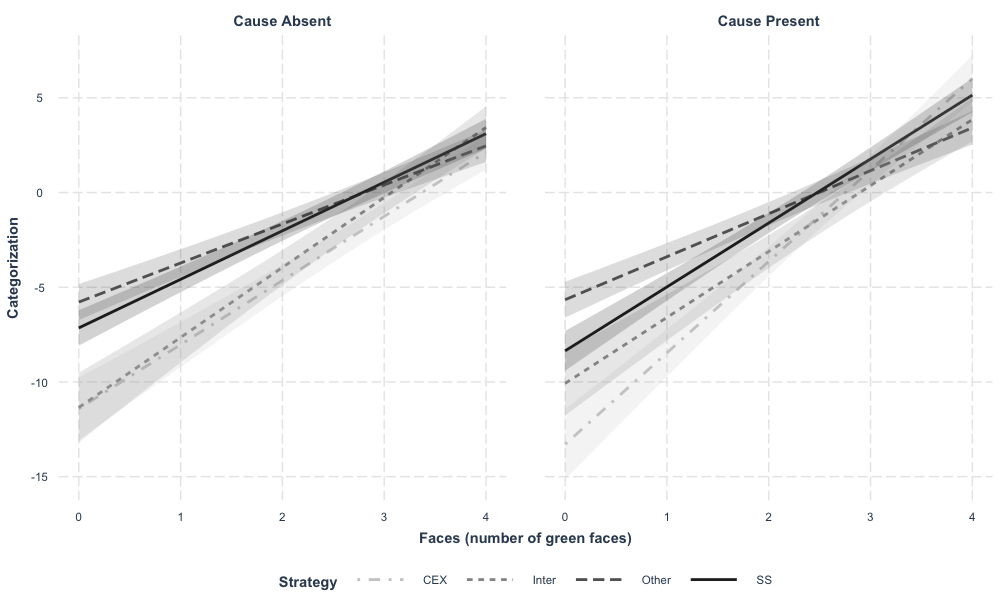


Figure A1. Interaction plot from Study 1 of the three-way interaction Strategy X Cause X Faces (computed using interactions package for R). Confidence intervals are represented. *Note*: *CEX = Counterexample, Inter = Intermediate, SS = Statistical.*

**Study 2**

**Categorization of trials**

Results from the Generalized mixed model with Categorization as the dependent variable, Strategy, Cause and Face along the interactions between those terms as independent variables showed a significant main effect of Strategy and Faces. In addition, there were significant interactions involving Strategy X Ratio and Cause X Ratio X Strategy.

Analysis of the main effect of Strategy showed that levels of categorization were similar between Counterexample reasoners (*EMM =* 0.009, *SE* = 0.004) and Intermediate reasoners (*EMM* = 0.02, *SE* = 0.009) which were significantly lower than Statistical (*EMM* = 0.07, *SE* = 0.02) and Other reasoners (*EMM* = 0.12, *SE* = 0.04).

Analysis of the Strategy X Faces interaction showed that the correlation between Categorization and Faces was significantly higher for Counterexample reasoners (*b* = 3.40, *SE* = 0.20) than for Intermediate (*b* = 2.52, *SE* *=* 0.20*)* , Statistical (*b* = 2.91, *SE*  = 0.15) and Other (*b* = 1.98, *SE* = 1.98, *SE =* 12.6) reasoners. Also, the correlation was significantly higher for Statistical reasoners than Intermediate and Other reasoners. Finally, the correlation was higher for Intermediate reasoners than for Other reasoners.

Analysis of the three-way interaction (see Figure A2) showed that when the putative cause was absent, the correlation between Categorization and the number of green Faces was higher for Counterexample (*b* = 3.07, *SE* = 0.23) and Statistical reasoners (*b =* 2.66, *SE* = 0.18) than for Intermediate (*b* = 2.06, *SE =* 0.22) and Other reasoners (*b* = 1.95, *SE* = 0.17). When the putative Cause was present, the correlation between the number of green Faces and Categorization was higher for Counterexample reasoners (*b* = 3.73, *SE* = 0.27) than for Intermediate (*b* = 2.97, *SE* = 0.29), Statistical (*b* = 3.15, *SE*  = 0.20) and Other reasoners (*b* = 2.01, *SE* = 0.17). This correlation was similar between Intermediate and Statistical reasoners, and higher for both Statistical and Intermediate reasoners than for Other reasoners.

We then computed pairwise comparisons (Wilcoxon tests, Holm’s correction) between strategies for each level of Faces and Cause.

For trials with 0 green faces, both when the putative cause was present absent, categorization of trials as patient cured were similar between strategies. The same pattern of results was observed for trials with 1 green face, when the putative cause was absent. When the putative was present, Intermediate, Counterexample and Statistical reasoners classified similarly trials as patient cured. Statistical and Counterexample reasoners classified less often trials as patient cured than Other participants.

For trials the 2 green faces when the cause was absent, levels of categorization were similar between strategies. However, when the putative cause was present, Counterexample reasoners classified less trials as patient cured than Other participants. Other comparisons were not significant.

For truly ambiguous trials, when the putative cause was present, Counterexample and Intermediate reasoners had similar levels of categorization. For both these strategies categorization of trials was lower then for Statistical reasoners and Other participants. Statistical reasoners and Other participants had similar levels of categorization. When the putative cause was absent, Counterexample reasoners classified less often trials as patient cured than Statistical reasoners and Other participants. Intermediate reasoners classified less often than Other participants trials as patient cured, but similarly to Statistical reasoners. Statistical reasoners and Other participants had similar levels of categorization.

For trials with four green Faces, when the cause was absent, Intermediate reasoners and Counterexample reasoners had similar levels of categorization of trials. Both categorized less often trials as patient cured than Statistical reasoners. Also, Intermediate reasoners had lower levels of categorization then Other reasoners. However, Counterexample and Other participants classified similarly trials. The same pattern was found for Other participants and Statistical reasoners. When the putative cause was present, both Counterexample and Intermediate reasoners classified less often trials as patient cured than Statistical reasoners. Other comparisons were not significant.

For trials with five green faces, when the putative cause was absent, the only significant difference was between Intermediate reasoners and Statistical reasoners. Other results were not significant. When the putative cause was present, classification of trials was similar between strategies.

Finally, for trials with six green faces, categorization was similar between strategies both when the putative cause was present and absent.

Table A.2. Mean levels of categorization of trials as patient cured as a function the Face, Cause and Strategy in Study 2.

| Face | Cause | CEX | Inter | Other | SS |
| --- | --- | --- | --- | --- | --- |
| 0 | A | 0.00000 (0.0000) | 0.02151 (0.0832) | 0.00654 (0.0467) | 0.00300 (0.0316) |
|  | P | 0.00000 (0.0000) | 0.01075 (0.0599) | 0.01307 (0.0653) | 0.00000 (0.0000) |
| 1 | A | 0.00000 (0.0000) | 0.00000 (0.0000) | 0.01961 (0.0980) | 0.01351 (0.0814) |
|  | P | 0.00000 (0.0000) | 0.01613 (0.0898) | 0.06863 (0.1738) | 0.00901 (0.0668) |
| 2 | A | 0.00565 (0.0434) | 0.02151 (0.0832) | 0.04575 (0.1337) | 0.01802 (0.0881) |
|  | P | 0.00565 (0.0434) | 0.00000 (0.0000) | 0.08497 (0.1982) | 0.02703 (0.1019) |
| 3 | A | 0.04068 (0.1440) | 0.03871 (0.1145) | 0.17843 (0.2532) | 0.10450 (0.2015) |
|  | P | 0.03559 (0.1483) | 0.01935 (0.0749) | 0.19412 (0.2709) | 0.11261 (0.2166) |
| 4 | A | 0.39548 (0.4526) | 0.25806 (0.4010) | 0.52288 (0.4282) | 0.58859 (0.4215) |
|  | P | 0.49718 (0.4606) | 0.44086 (0.4744) | 0.57516 (0.4273) | 0.69670 (0.4034) |
| 5 | A | 0.69492 (0.4257) | 0.58065 (0.4672) | 0.81373 (0.3600) | 0.81982 (0.3554) |
|  | P | 0.76271 (0.4189) | 0.72581 (0.4442) | 0.83333 (0.3697) | 0.87838 (0.3178) |
| 6 | A | 0.93220 (0.2298) | 0.90323 (0.2748) | 0.93464 (0.2002) | 0.97598 (0.1248) |
|  | P | 0.98870 (0.0608) | 1.00000 (0.0000) | 0.98693 (0.0653) | 1.00000 (0.0000) |


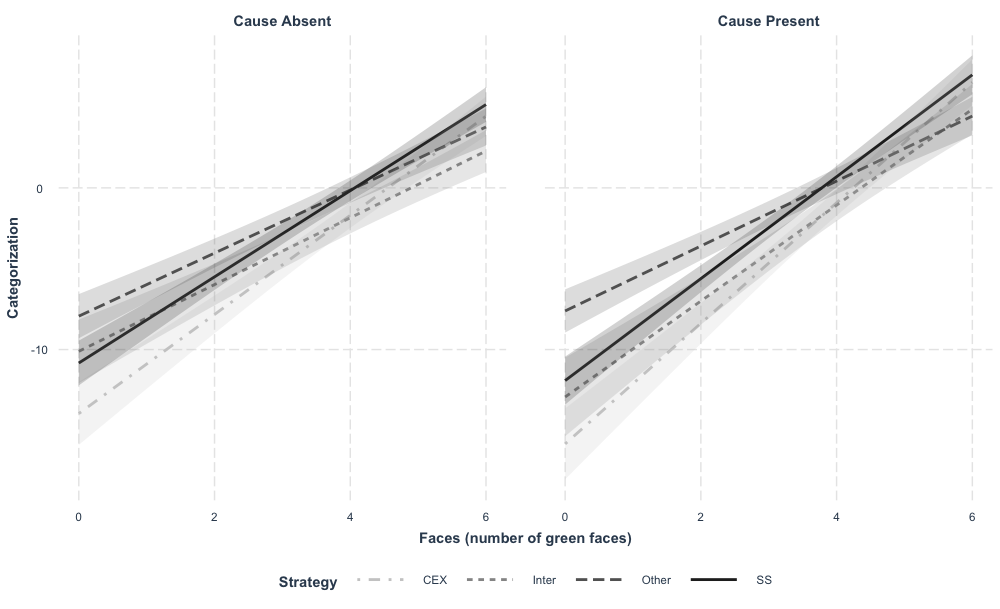


Figure A2. Interaction plot from Study 2 of the three-way interaction Strategy X Cause X Faces (computed using interactions package for R). *Note*: *CEX = Counterexample, Inter = Intermediate, SS = Statistical.*
